# Characterisation of a putative AraC transcriptional regulator from *Mycobacterium smegmatis*

**DOI:** 10.1016/j.tube.2014.08.007

**Published:** 2014-12

**Authors:** Dimitrios Evangelopoulos, Antima Gupta, Nathan A. Lack, Arundhati Maitra, Annemieke M.C. ten Bokum, Sharon Kendall, Edith Sim, Sanjib Bhakta

**Affiliations:** a Mycobacteria Research Laboratory, Institute of Structural and Molecular Biology, Department of Biological Sciences, Birkbeck, University of London, Malet Street, London WC1E 7HX, UK; b Department of Pharmacology, University of Oxford, Mansfield Road, Oxford OX1 3QT, UK; c Department of Pathology and Infectious Diseases, The Royal Veterinary College, Royal College Street, London NW1 0TU, UK

**Keywords:** AraC, Transcriptional regulator, Mycobacteria, Mycobacterial two-hybrid system, *nat* operon, Protein–protein interaction

## Abstract

*MSMEG_0307* is annotated as a transcriptional regulator belonging to the AraC protein family and is located adjacent to the arylamine *N*-acetyltransferase (*nat*) gene in *Mycobacterium smegmatis*, in a gene cluster, conserved in most environmental mycobacterial species. In order to elucidate the function of the AraC protein from the *nat* operon in *M. smegmatis*, two conserved palindromic DNA motifs were identified using bioinformatics and tested for protein binding using electrophoretic mobility shift assays with a recombinant form of the AraC protein. We identified the formation of a DNA:AraC protein complex with one of the motifs as well as the presence of this motif in 20 loci across the whole genome of *M. smegmatis*, supporting the existence of an AraC controlled regulon. To characterise the effects of AraC in the regulation of the *nat* operon genes, as well as to gain further insight into its function, we generated a Δ*araC* mutant strain where the *araC* gene was replaced by a hygromycin resistance marker. The level of expression of the *nat* and *MSMEG_0308* genes was down-regulated in the *ΔaraC* strain when compared to the wild type strain indicating an activator effect of the AraC protein on the expression of the *nat* operon genes.

## 1 Introduction

Transcriptional factors modulate gene expression through binding to a specific DNA sequence usually found upstream of the gene or the genomic area that they control. They are important proteins that can help cells acclimatise to challenging environments based on the changing external stimuli. The AraC/XylS protein family of transcriptional regulators, present in bacterial species is involved in a variety of cellular processes from carbon metabolism to stress responses and the regulation of virulence [1]. Common characteristics of the AraC proteins is the presence of a conserved region of 100 residues in the C-terminal region of the protein that form a helix-turn-helix structure responsible for DNA binding, a second region in the *N*-terminal region of the protein contains a ligand binding domain and a peptide-linker region connecting the two functional domains. The proteins that belong to the AraC/XylS family usually recognise palindromic DNA sequences and bind to them by forming dimers using the helix-turn-helix domain [2].

*Mycobacterium tuberculosis*, the causative agent of tuberculosis (TB) can survive within macrophages as well as in the extreme environment found in granulomas during infection in the human body. For this reason, the genome of *M. tuberculosis* contains an exceptionally large number of transcriptional factors, including 13 sigma factors, 5 anti-sigma factors and 7 anti-anti-sigma factors [3] which assist its adaptation to different environments and stresses. Six of these 190 transcription factors belong to the AraC/XylS family (Rv1317, Rv1395, Rv1931c, Rv3082c, Rv3736 and Rv3833). Most of the *M. tuberculosis* AraC proteins characterised, such as the AraC proteins encoded by Rv1395, Rv1931c and Rv3082c genes are linked with virulence as their genetic alteration generates an attenuated phenotype either *in vitro* in macrophage infection model or *in vivo* in mice [4], [5], [6], [7], [8]. Until now there has been no functional information regarding the role of the Rv3736 and Rv3833 encoded AraC proteins in *M. tuberculosis*. Furthermore, *Mycobacterium smegmatis*, the saprophytic environmental species of the genus *Mycobacterium* and a common laboratory surrogate for molecular genetic studies of *M. tuberculosis*, contains 16 different AraC proteins encoded in its genome, indicating the need of this organism to adapt to multiple niches. One of these AraC proteins is encoded by the *MSMEG_0307*, gene which is located between the arylamine *N*-acetyltransferase (*nat*, *MSMEG_0306*) gene and a novel oxido-reductase (*MSMEG_0308*) that is believed to be involved in riboflavin biosynthesis [9].

The *nat* operon in *M. tuberculosis* has been validated as a likely therapeutic target due to its important endogenous roles in *M. tuberculosis*, related to cholesterol degradation, cell wall biogenesis, intracellular growth and altered drug susceptibility [10], [11], [12]. In *M. tuberculosis*, the *hsaA* (*Rv3570c*), *hsaB* (*Rv3567c*), *hsaC* (*Rv3568c*) and *hsaD* (*Rv3569c*) genes are co-transcribed with the *nat* (*Rv3566c*) gene and their corresponding proteins have been shown to be directly involved in the cholesterol metabolism pathway [13]. The NAT protein utilises acyl co-enzymeA (CoA) catabolites, including acetyl CoA and *n*-propionyl CoA. These intermediates play a central role in metabolic support of cell wall biosynthesis [14], [15]. Cholesterol is considered to be a vital energy source for *M. tuberculosis* cells growing within macrophages [16]. In addition, this *nat* gene cluster in *M. tuberculosis* is under the control of the *kstR* transcription factor (Rv3574) that controls a regulon of genes involved in lipid and cholesterol metabolism [17].

**Figure 1:**
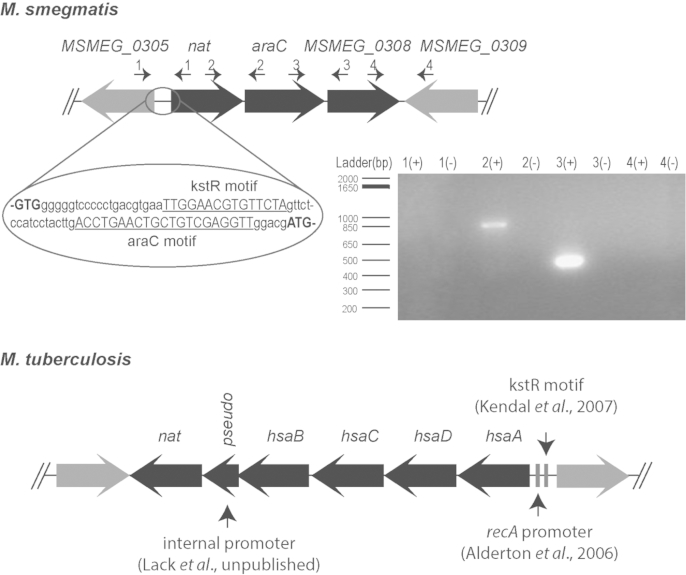
**The *nat* operon in *M. smegmatis***. A comparison between the *nat* operons in *M. smegmatis* and *M. tuberculosis*. In the *nat* operon of *M. smegmatis* the intergenic region between *nat* and *MSMEG_0305* genes is shown in loop with the location of the kstR and araC motifs highlighted in the sequence. In the *M. tuberculosis nat* operon, the identified promoter and kstR sequences are shown with the arrows. RT-PCR amplification of the overlapping regions of the *nat* operon genes using specific primers, shown as numbered arrows on top of the genes. Lanes with (+) on the agarose gel correspond to cDNA whereas lanes with (−) corresponds to negative control, i.e. cDNA that produced without the addition of reverse transcriptase (RT) in order to distinguish possible genomic DNA contamination. Primer set 1: MSMEG_0305-*nat*, primer set 2: *nat*-*MSMEG_0307*, primer set 3: *MSMEG_0307*-*MSMEG_0308* and primer set 4: *MSMEG_0308*-*MSMEG_0309*.

In contrast, the gene organisation around *nat* differs in *M. smegmatis* and the *hsaA*-*D* genes (*MSMEG_6035*- *MSMEG_6038*) are clustered together in a region about 57 kb downstream from the *nat* locus. Nevertheless, kstR regulatory DNA sequences are present between the *nat* and the *MSMEG_0305* genes (Figure 1) and upstream of the *hsaA*-*D* gene cluster in the genome of *M. smegmatis* indicating possible co-regulation. However, it has been shown previously that the *nat* gene cluster in *M. smegmatis* is not directly controlled by the *kstR* transcriptional regulator but rather the *MSMEG_0305* is under the effect of the KstR regulator [17], [18].

In the view of the presence of a transcription factor that belongs to the AraC protein family in the *nat* gene cluster in *M. smegmatis* it is important to assess whether it influences the expression of *nat* and the adjacent gene for the novel reductase (MSMEG_0308). The question of whether the AraC transcriptional factor has a role in the regulation of genes that are involved in lipid and cholesterol metabolism in *M. smegmatis* also needs to be addressed. We report here, the characterisation of the AraC-family transcriptional regulator MSMEG_0307 from *M. smegmatis* using biochemical assays, the characterisation of its regulon and DNA binding sites, as well as its influence on the regulation of gene expression of the *nat* gene cluster in *M. smegmatis*. In addition, we have characterised a protein–protein interaction network that is formed by the *nat* operon gene products.

## 2 Materials and methods

### 2.1 Bacterial strains, growth conditions and plasmids

The bacterial strains and plasmids used in this study are listed in Table S4. The oligonucleotide primers are listed in the Supplementary Table S1. *Escherichia coli* strains JM109 and BL21(DE3)pLysS were grown in Luria–Bertani (LB) broth with rotation at 200 rpm or in LB agar at 37 °C, unless specified otherwise. *M. smegmatis* mc^2^155 [19] and the modified strains were grown in Middlebrook 7H9 broth supplemented with 0.2% (v/v) glycerol, 0.05% (w/v) Tween-80 and 10% ADC (Albumin-Dextrose-Catalase, BD) with rotation at 180 rpm or in Middlebrook 7H10 agar supplemented with 0.5% (v/v) of glycerol and 10% OADC (Oleic acid-Albumin-Dextrose-Catalase, BD) at 37 °C, unless specified otherwise. Comparative growth curves of *M. smegmatis* mc^2^155 and the *Δnat*, *ΔMSMEG_0307* and *ΔMSMEG_0308* strains were performed using mycobacterial minimal medium (0.5 g l^−1^ of l-asparagine, 1 g l^−1^ of KH_2_PO_4_, 2.5 g l^−1^ of Na_2_HPO_4_, 50 mg l^−1^ of ferric ammonium citrate, 0.5 g l^−1^ of MgSO_4_.7H_2_O, 0.5 mg l^−1^ of CaCl_2_ and 0.1 mg l^−1^ of ZnSO_4_) supplemented with a carbon source (glycerol, glucose, melibiose or cholesterol) at 0.2% (v/v). Antibiotics were used at the following concentrations (μg ml^−1^): kanamycin (30), chloramphenicol (34), hygromycin B (100 for *E. coli* and 50 for *M. smegmatis*) and trimethoprim (12). All chemicals were purchased from Sigma–Aldrich (Poole, UK) unless otherwise stated. High-fidelity Phusion DNA polymerase (NEB) was employed in all cloning related PCR reactions whereas *Taq* DNA polymerase (NEB) was used in all other PCR reactions in this study. Restriction digestion enzymes were purchased from New England Biolabs (Hitchin, UK). All constructs were confirmed by sequencing (Gene Service, UCL).

### 2.2 Identification promoter sites and DNA motifs

All mycobacterial genome sequences that were used in this study were obtained from the NCBI (http://www.ncbi.nlm.nih.gov). BLAST analysis was performed using the NCBI BLAST algorithm [20]. Multiple sequence alignments were done using ClustalW algorithm at the EBI server [21]. For viewing, annotating and comparing mycobacterial genomes, the java-based software packages Artemis and ACT, from the Sanger Institute, were used [22], [23]. The sequence viewer, PromView (http://www.comlab.ox.ac.uk-/activities/compbio/bioinformatics-/software/index-.htm#PromView) was used for the search of consensus promoter sequences obtained from previously published mycobacterial promoters [24]. The MEME algorithm [25] was used to discover conserved palindromic DNA motifs among mycobacterial species and MAST [26] was used to identify the presence of these motifs in the genome of *M. smegmatis* mc^2^155.

### 2.3 Cloning, overexpression and purification of MS0307

The gene encoding the AraC protein (*MSMEG_0307*) from *M. smegmatis* mc^2^155 was PCR amplified from genomic DNA and cloned into pET28b (+) vector (Novagen). The *N*- terminus of the AraC-family transcriptional regulator MSMEG_0307 was co-transcribed with a thrombin cleavage site followed by a hexa-histidine tag. The recombinant AraC-family transcriptional regulator MSMEG_0307 was produced in BL21(DE3)pLysS cells at 18 °C following induction with 0.5 mM IPTG overnight. Cells were lysed by sonication on wet ice (5 cycles of 45sec on, 45sec off) and the AraC-family transcriptional regulator MSMEG_0307 was purified using nickel affinity chromatography (Invitrogen). The His-tagged MSMEG_0307 protein was then further purified on a HiLoad 16/60 Superdex™ 75 pg (Pharmacia) preparative gel filtration column, equilibrated with 20 mM Tris–HCL pH 8, 100 mM NaCl. Fractions contained pure (>99%) His-tagged MSMEG_0307 protein was pooled and concentrated using an Amicon Ultra concentrator (Millipore) at 5 mg ml^−1^ and stored in 50% (v/v) glycerol in −80 °C for further use.

### 2.4 Electrophoretic mobility shift assays (EMSAs)

The DNA fragments (∼300bp) containing the binding sequence motifs were amplified using PCR and primers (Table S1) and further purified. The reactions had a final volume of 10 μl and contained 100 ng of DNA, 1× EMSA buffer (20 mM Tris.HCl pH8, 75 mM NaCl, 10 mM MgCl_2_) and increasing concentrations of recombinant His-tagged MSMEG_0307 protein (0.01 μg to 1 μg). The reactions were incubated at room temperature for 30 min and then were loaded onto a 5% (v/v) native polyacrylamide gel. Following electrophoresis the gels were stained with ethidium bromide and the bands were visualised using BioDoc-It™ imaging system (UVP, Cambridge, UK).

### 2.5 Generation of *ΔaraC* in *M. smegmatis* and complementation studies

The deletion of the *MSMEG_0307* gene (*MSMEG_0307*) from *M. smegmatis* mc^2^155 was performed using the method of specialised transduction as described previously [27]. Briefly, the left (898 bp) and right (782 bp) arms of the *MSMEG_0307* gene were PCR amplified using the primers given in table S1 and cloned into the suicide delivery vector p0004S to create the allelic-exchange plasmid p0004S-*MSMEG_0307*. The p0004S- *MSMEG_0307* was then *Pac*I digested and packed into the temperature sensitive mycobacteriophage phAE159 to generate the allelic-exchange phage phΔaraC. Wild-type *M. smegmatis* mc^2^155 was transduced using high-titre phΔ*MSMEG_0307* phages as described [27]. Following the specialised transduction, hygromycin resistant colonies were screened by PCR using a gene internal and external primer set (Table S1) and Δ*araC* mutants were further confirmed by DNA sequencing. Complementation of the Δ*MSMEG_0307* as well as overexpression of the WT *M. smegmatis* mc^2^155 were performed using the pMV261 plasmid [28] with the native MSMEG_0307 expressed under hsp60 mycobacterial promoter located in the plasmid.

### 2.6 Drug susceptibility assay

The susceptibility of mycobacterial strains against various antibiotics was determined using the resazurin redox indicator assay as described previously [29]. Briefly, wild-type *M. smegmatis* mc^2^155 and the Δ*MSMEG_0307* mutant were grown until mid-exponential phase (1 OD_600_) and then 100 μl of diluted cells (10^4^ CFUs) were added into a 96 well plate that contained 100 μl of two-fold dilutions of antibiotics at various concentrations in μg/mL [isoniazid (INH 50 to 0.09), pyrazinamide (PZA 50 to 0.09), rifampicin (RMP 50 to 0.09), ethambutol (EMB 150 to 0.29), streptomycin (STM 50 to 0.09), kanamycin (KAN 50 to 0.09), ampicilin (AMP 150 to 0.29) and chloramphenicol (CLP 50 to 0.09)]. The plates were incubated at 37 °C for 2 days. Following 24 h of incubation, 50 μl of 0.01% (w/v) sterile resazurin solution in presence of 1% (v/v) Tween-80 was added to all wells of the plate and left overnight at 37 °C. The minimum inhibitory concentrations (MICs) were defined as the lowest antibiotic concentration of the well where bacterial cells were not able to grow and thus did not reduce the resazurin dye.

### 2.7 RNA extraction and cDNA synthesis

Wild-type *M. smegmatis* mc^2^155, *ΔMSMEG_0307*, wild-type *M. smegmatis* mc^2^155 with either the empty vector (pMV261) or overexpressing *MSMEG_0307* (pMV*araC*) and the *ΔMSMEG_0307* complimented mutant cells *were grown in Middlebrook 7H9 broth until OD_600_ was 0.8* and total RNA was extracted from using the GTC method as previously described [17] and cDNA was synthesised using SuperScript III Reverse Transcriptase (Invitrogen) according to the manufacturer's instructions. A control was set up to assess genomic DNA contamination by replacing the Superscript III Reverse Transcriptase with water.

### 2.8 Operon analysis

The boundaries of the *nat* operon were identified by PCR amplification of the intergenic regions of the operon and adjacent genes using *M. smegmatis* mc^2^155 cDNA as a template and specific primers (Table S1) for each region. Positive (gDNA from *M. smegmatis* mc^2^155) and negative controls (cDNA made without Reverse Transcriptase) were also used. The amplicons were then analysed using agarose gel electrophoresis.

### 2.9 RT-qPCR

Real-time quantitative polymerase chain reactions (RT-qPCR) were performed using the DyNAmo SYBR Green qPCR kit (NEB) on the MJ Research Bio-Rad Real Time PCR Opticon Engine 2 System (GRI). *M. smegmatis* mc^2^155 gDNA was used for the generation of a standard curve and *sigA* gene (*MSMEG_2758*; a mycobacterial sigma factor) was used as a reference gene for the relative quantification method. Briefly, a 20 μl reaction was set up on ice containing 1× DNA Master SYBR Green I mix, 1 μl of cDNA and 0.3 μM of each primer (Table S1). The PCR reactions were initially heated to 95 °C for 10 min before 35 cycles of 95 °C for 30 s, 62 °C for 20 s, and 72 °C for 20 s were performed. Fluorescence was measured at the end of each cycle following a heating step to 80 °C to ensure the denaturation of any primer-dimers. At the end of the PCR, melting curve analysis was performed to verify the product specificity. The experiment was performed in duplicate and each gene was measured in triplicates (three biological replicates, two experimental replicates) giving a total of six data points per gene. Fold changes were calculated using the 2^−ΔΔCt^ statistical method [30].

## 3 Results

### 3.1 Defining the *nat* operon in *M. smegmatis*

In order to define the presence and extent of a *nat* operon in *M. smegmatis*, RT-PCR analysis was performed on the basis that any amplicons obtained using intergenic primers would indicate that the two genes are co-transcribed together and thus belong to the same operon (Figure 1). Using this rationale, we obtained amplicons for the intergenic regions between *nat*-*MSMEG_0307* (*MSMEG_0306*-*MSMEG_0307*) and *MSMEG_0307*-*MSMEG_0308* (*MSMEG_0307*-*MSMEG_0308*) but not between the *MSMEG_0305*-*nat* and *MSMEG_0308*-*MSMEG_0309*, indicating that in *M. smegmatis* the *nat* operon consists of three genes (Figure 1). Using bioinformatic analyses, we search for a putative promoter sequence using as input known mycobacterial promoters; however we were not able to identify a conserved promoter sequence upstream of *nat* gene at the start of the operon. A comparative analysis on different mycobacterial genomes indicated that a similar gene organisation was seen in the *nat* operon among fast-growing mycobacteria as opposed to the *nat* and *hsaA-D* clusters found in slow-growing mycobacteria (Figure S1).

**Figure 2:**
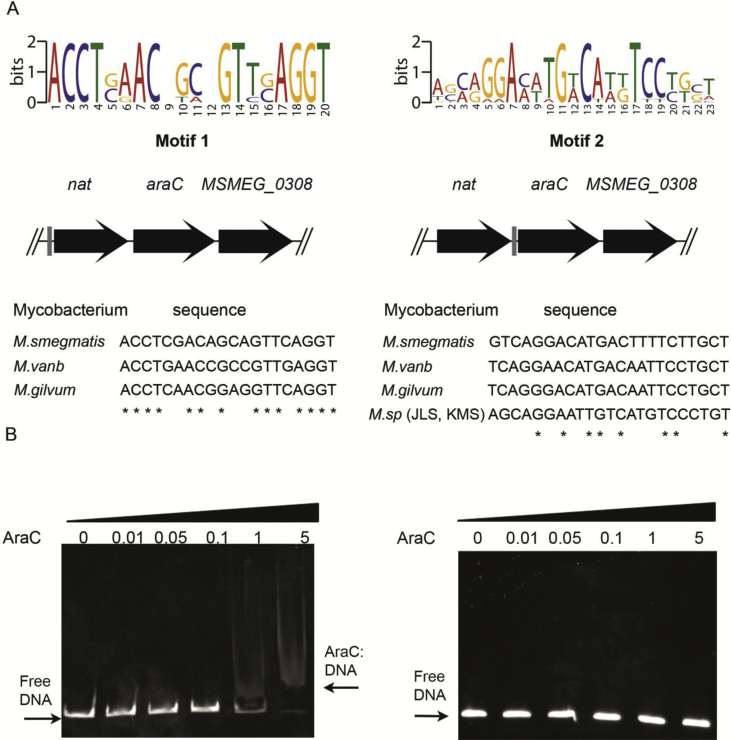
**Investigation of DNA regulatory motifs found in the *nat* operon in *M. smegmatis***. (A) The palindromic DNA sequences are shown on the graphs. The big letters illustrate the consensus nucleotides in the motifs. The grey boxes in the operon genes below the motifs show the positions of the motifs in the relationship to the operon. The nucleotide sequences of each of the motifs in different mycobacterial genomes are shown with the consensus nucleotides highlighted with an asterisk (*). (B) EMSA assays with MSMEG_0307 recombinant protein and the identified motifs. DNA containing the Motif 1 or the Motif 2 were incubated with increasing concentration of MSMEG_0307 protein [0, 0.01, 0.05, 0.1, 1 and 5 μg/mL] and then were loaded onto an 8% polyacrylamide gel.

Thorough sequence analysis using the MEME algorithm [25] was carried out on the *M. smegmatis nat* operon as well as a 1.5 kb DNA fragment upstream of the *nat gene* in order to identify the presence of regulatory DNA sequences that could be affected by the *MSMEG_0307* gene product. Comparative genome analyses were performed on the closely related fast-growing mycobacterial species that have similar *nat* gene clusters and an *MSMEG_0307* orthologue (*M. smegmatis*, *Mycobacterium gilvum*, *Mycobacterium vanbaalenii* and *Mycobacterium* sp. MCS/KMS/JLS). This approach identified two different regulatory motifs. The motif designated from now on as Motif 1 is located between −25 and −5 upstream of the *nat* gene start codon (Figure 2A) and another motif designated from now on as Motif 2 is located between −57 and −34 upstream of the *MSMEG_0307* gene (Figure 2A). Motif 1 consists of a 20 bp palindromic DNA sequence (**ACCTCGACAGCAGTTCAGGT**) (Figure 2A) and the Motif 2 consists of a 23 bp DNA sequence (**GTCAGGACATGACTTTTCTTGCT**) (Figure 2A). In order to identify the presence of these two motifs elsewhere in the genome of *M. smegmatis* as well as possible sites of action of the MSMEG_0307 protein, the MAST algorithm was employed to search a database of intergenic regions of the *M. smegmatis* genome [26]. Motif 1 was found to be present in twenty additional instances in the *M. smegmatis* genome (Table S2) and the Motif 2 in five additional instances (Table S3). This indicates the existence of a regulon controlled by the AraC transcription regulator MSMEG_0307.

### 3.2 Binding of AraC_Msmeg_ to the identified DNA motifs

In order to determine whether the MSMEG_0307 protein binds to any of the two identified motifs, the MSMEG_0307 protein from *M. smegmatis* mc^2^155 was cloned, over-expressed and purified as a recombinant protein (Figure S2). DNA fragments containing Motif 1 or 2 were amplified using PCR and used for Electrophoretic Mobility Shift Assays (EMSA). As shown in Figure 2B the presence of recombinant MSMEG_0307 was able to produce a band shift indicating the binding of the protein to Motif 1. Interestingly, in a parallel experiment no DNA band shift was observed when Motif 2 was used as a substrate for the MSMEG_0307 (Figure 2B) indicating that the MSMEG_0307 protein binds specifically to the Motif 1.

### 3.3 Characterisation of a Δ*araC* strain

In order to investigate the role of the MSMEG_0307 gene product of the *nat* operon in *M. smegmatis* the gene (*MSMEG_0307*) was deleted from the genome using specialised transduction [27]. Resistant colonies were screened for the presence of the deletion by PCR using one set of primers which amplified either an internal region of the gene or a region flanking the deleted *MSMEG_0307* gene. As expected, the *ΔMSMEG_0307* strain did not show any PCR amplification using the internal set and had an amplicon with a size difference using the external primer set indicating the presence of the hygromycin resistance cassette in the genome in the place of the *MSMEG_0307* gene (Figure S3). A similar methodology was applied for the generation of the *MSMEG_0308* gene deletion mutant (*ΔMSMEG_0308*) as well.

**Figure 3:**
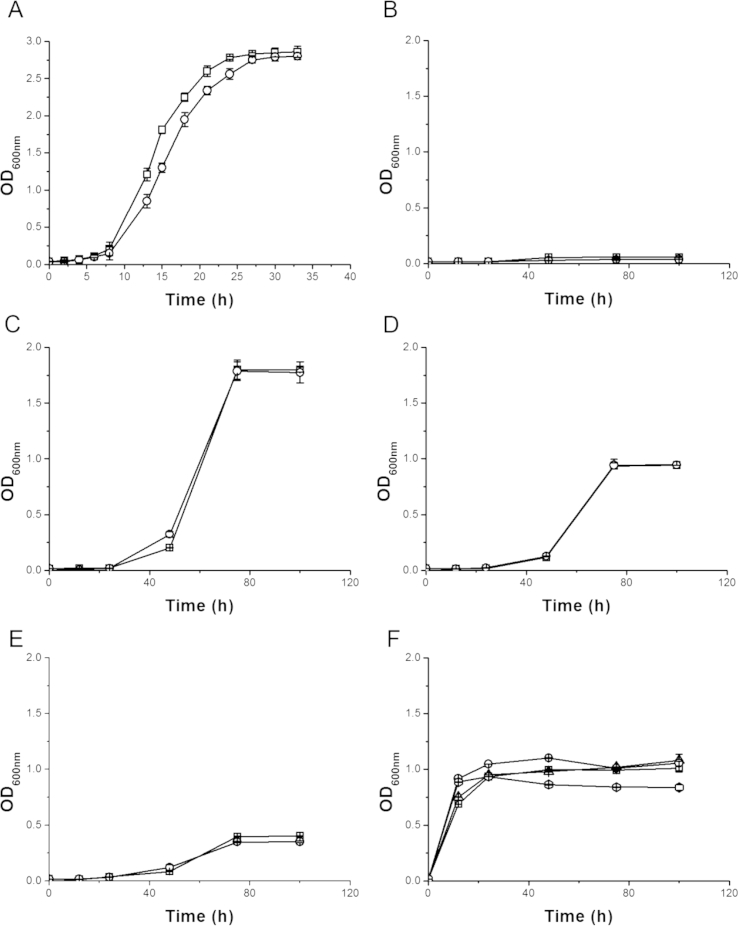
**Growth curves of WT and *Δ****MSMEG_0307****M. smegmatis* in enriched and minimal media**. The cultures were inoculated with the same amount of bacteria (∼10^7^) and the growth was detected by measuring the OD_600nm_ over time. The volume of each culture was 100 mL. Growth curves of WT (□), *Δnat* (▵), *ΔMSMEG_0307* (○) and *ΔMSMEG_0308* (◊) *M. smegmatis* in (A) Middlebrook 7H9medium, (B) minimal medium with no added carbon source (C) minimal medium with glycerol (D) minimal medium with glucose (E) minimal medium with melibiose and (F) minimal medium supplemented with cholesterol.

The ability to generate a *ΔMSMEG_0307* strain indicates that the *MSMEG_0307* gene is not essential for *in vitro* growth of *M. smegmatis*. Comparative growth curves in enriched media between wild type and the *ΔMSMEG_0307* strain indicated that the loss of the *MSMEG_0307* gene did not significantly affect the growth of the mutant strain (Figure 3A). Subsequently the ability of the *ΔMSMEG_0307* strain to grow in minimal media with glycerol, glucose, melibiose [31] was tested as several bacterial AraC transcription factors are known to control genes that are responsible for the degradation of complex carbon sources, such as arabinose metabolism in *E. coli* [32]. It was found that the deletion of the MSMEG_0307transcription regulator had little effect on the growth on these carbon sources (Figure 3, Figure 3B–E).

As the genes of the *nat* operon in *M. tuberculosis* are involved in cholesterol metabolism [13], [33], the ability of the *ΔMSMEG_0307* as well as *Δnat* [34] and *ΔMSMEG_0308* strains to grow in presence of cholesterol as the sole carbon source was also examined. The results showed that the proteins encoded by *nat* operon in *M. smegmatis* are not essential for degradation of cholesterol (Figure 3F) in contrast to those present in *M. tuberculosis*.

Furthermore, in order to investigate the possibility that the MSMEG_0307regulator is involved in the development of multidrug resistance as other studied AraC proteins are known to be [35], we tested the susceptibility of the *ΔMSMEG_0307* strain to a variety of antibiotics including all the first line anti-tuberculosis drugs (isoniazid, ethambutol, rifampicin and pyrazinamide). The susceptibility pattern of the two strains, WT and *ΔMSMEG_0307*, was similar apart from negligible differences observed in the MIC values on rifampicin, kanamycin and chloramphenicol (Figure S4).

### 3.4 MSMEG_0307 protein regulates the expression of the *nat* operon in *M. smegmatis*

**Figure 4:**
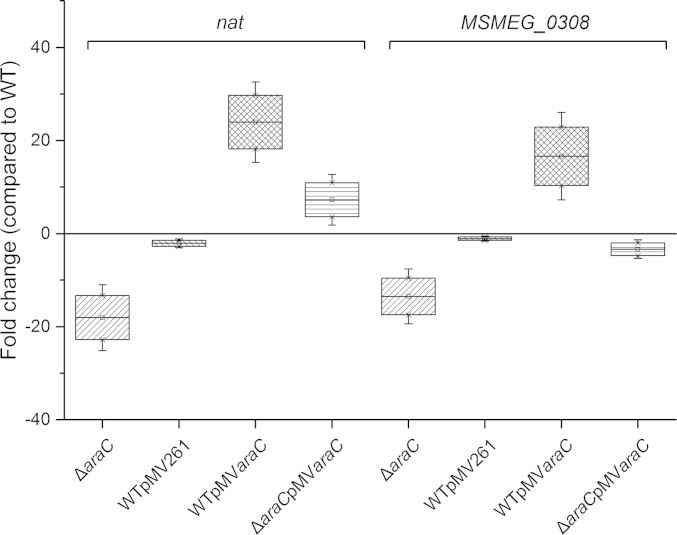
**Differential expression of *nat* and *MSMEG_0308* genes in *M. smegmatis* strains**. The fold changes of the *ΔMSMEG_*0307, WT containing the empty pMV261 vector (WTpMV261), WT overexpressing *MSMEG_0307* (WTpMV*araC*) and the complementation strain *ΔMSMEG_*0307 containing the pM*araC* vector (WTpMV*araC*) compared to WT. Relative quantification of the gene expression levels were applied using the *ΔΔCt* statistical method and the expression of *sigA* gene from *M. smegmatis* was used as reference gene. The range of the box plots represent the SE with the mean outlined as the small box inside each box plot. Confidence intervals at 1% and 99% are mentioned by the x symbols at each box.

As the recombinant MSMEG_0307, a putative transcription regulator, was able to bind specifically to the Motif 1 upstream of the *nat* operon in *M. smegmatis*, the effect of the *MSMEG_0307* gene deletion on the expression of the other genes of the operon was studied using RT-qPCR. The comparison of the relative expression levels of *nat* (*MSMEG_0306*) and *MSMEG_0308* genes between the WT and the *ΔMSMEG_0307* mutant revealed that the deletion of the *MSMEG_0307* gene has a significant effect on the expression of the other operon genes (Figure 4) suggesting that the AraC protein controls the gene expression of the operon. Both *nat* and *MSMEG_0308* genes were down-regulated in the *ΔMSMEG_0307* mutant by 18 and 13 fold respectively indicating that the AraC transcription factor acts as an activator for the expression of *nat* and *MSMEG_0308* genes. On the contrary, overexpression of the *MSMEG_0307* gene in the wild-type *M. smegmatis* from the pMV*araC* plasmid increased the expression of both *nat* and *MSMEG_0308* by 23 and 16 fold respectively (Figure 4) confirming the activator effect of the MSMEG_0307 protein on the operon. The overexpression of the *MSMEG_0307* gene using the pMV*araC* construct in *M. smegmatis* was also confirmed by qPCR (data not shown). Complementation of the *ΔMSMEG_0307* strain with the same plasmid (pMV*araC*) was able to compensate for the down regulation of the *nat* gene resulting in a 7 fold up regulation; however, this was not the case for the *MSMEG_0308* gene that remained down regulated 3 fold. The presence of the internal cassette in place of *MSMEG_0307* gene might partially explain the reason why *MSMEG_0308* gene expression remained down regulated in the complemented *ΔMSMEG_0307* strain.

## 4 Discussion

The regulation of gene expression is essential for all living organisms to adapt to various environmental and physiological conditions and stresses. In this study, we describe the role of a transcriptional regulator from the AraC/XylS family in *M. smegmatis*, a fast growing, environmental species of *Mycobacterium*. We have identified that the AraC-family transcriptional regulator MSMEG_0307 protein recognises and binds to a palindromic motif upstream of the *nat* operon in *M. smegmatis* and that it is also involved in the regulation of the expression of the *nat* and the *MSMEG_0308* genes of the same operon. It is also very likely that it auto-regulates its own expression as other AraC proteins are known to do [36], [37] and thus controls the gene expression of the *nat* operon in *M. smegmatis*. In addition, the palindromic DNA sequence that the MSMEG_0307 binds to, has been found in 20 loci across the genome and this might represent an early view of the regulon that is under the control of the MSMEG_0307 protein.

The exact roles of these 20 genes that might be under the control of the MSMEG_0307 regulator have not been fully assigned; however, they can be divided into five main categories based on their function excluding the 6 hypothetical proteins (Table S2). There are four genes encoding for ATP-binding cassette (ABC) transporters and two transmembrane proteins with unknown functions. ABC transporters that might be under the control of AraC constitute universal transporter systems that are responsible for the transfer of a variety of substrates across the cell membrane [38]. In addition, one of the mechanisms of antibiotic resistance in bacteria is due to specific ABC transporters. The fluoroquinolone efflux pump encoded by the Rv2686c-Rv2688c operon in *M. tuberculosis* is an example [39]. Four of the genes that might be controlled by the MSMEG_0307 protein, encode proteins that are involved in metabolic pathways, including the NAT protein that has been shown to be involved in lipid biosynthesis [10] and a cholesterol degradation pathway [14]. In addition, there are three genes involved in oxidation/reduction reactions and three more that are involved in the regulation of gene expression. One of these 20 genes encodes an alternative sigma factor (*MSMEG_0574*) *rpoE1*, a putative extracytoplasmic (ECF) function alternative sigma factor. *M. tuberculosis* contains 10 copies of ECF alternative sigma factors and they are believed to act in a similar manner to the two component system enhancing the adaptation of bacteria under different physiological stages and pathogenesis [40]. It is clear that the binding of the MSMEG_0307 protein to the conserved Motif 1 located upstream of the *nat* gene has a direct effect on the expression of *nat* and *MSMEG_0308* transcripts.

Our preliminary studies using protein-fragment complementation [41] revealed the presence of a small protein complex made from the gene products of the *nat* operon (Figure S5). We also hypothesise that the MSMEG_0307, transcription factor, might also interact with sigma factors as parts of the RNA polymerase in order to support the initiation of the transcription on this genomic area.

Although there is no clear evidence of an orthologue of the MSMEG_0307 AraC protein in *M. tuberculosis* it is clear from comparative genomic analyses that all fast-growing environmental mycobacteria sequenced to date possess a similar gene architecture in their *nat* gene clusters and that the AraC-family transcriptional regulator MSMEG_0307 protein and its preferred DNA binding motif are highly conserved, suggesting that this genomic area plays an important role in the adaptation of these mycobacterial species to their specific environment. It will be interesting to identify the external stimuli as well as the ligands that bind to the MSMEG_0307 protein. Furthermore, ascertaining the biological significance of the MSMEG_0307 protein being situated next to NAT in environmental mycobacteria and providing an explanation of the different evolutionary pathways adopted by fast-growing environmental and slow-growing pathogenic mycobacteria, will give us an insight into the unique characteristics of the adaptation of this genus to multiple environments. This is the first report of the role of the AraC-family transcriptional regulator MSMEG_0307 protein from the *M. smegmatis nat* operon.
