# Supplementary material for: Characterisation of a putative AraC transcriptional regulator from Mycobacterium smegmatis
Source: Tuberculosis (Edinb). 2014 Dec;94(6):664–71. doi: 10.1016/j.tube.2014.08.007 (PMC4266540; doi:10.1016/j.tube.2014.08.007)
Supplement: Supplementary file 2 [file mmc2.docx]

**SUPPLEMENTARY INFORMATION**

**Title: Characterisation of a putative AraC transcriptional regulator from *Mycobacterium smegmatis***

Dimitrios Evangelopoulos^a b 1^, Antima Gupta^a 2^, Nathan A. Lack^b 3^, Arundhati Maitra^a^, Annemieke M.C. ten Bokum^c 4^, Sharon Kendall^c^, Edith Sim^b 5^ and Sanjib Bhakta^a✍^

*Author affiliations:*

^a^Mycobacteria Research Laboratory, Institute of Structural and Molecular Biology, Department of Biological Sciences, Birkbeck, University of London, Malet Street, London WC1E 7HX, UK.

^b^Department of Pharmacology, University of Oxford, Mansfield Road, Oxford OX1 3QT, UK.

^c^Department of Pathology and Infectious Diseases, The Royal Veterinary College, Royal College Street, London NW1 0TU, UK.

*Present addresses:*

^1^Centre for Clinical Microbiology, University College London, Royal Free Campus, Rowland Hill Street, London NW3 2PF, UK.

^2^Centre for Systems and Synthetic Biology (CSSB), Heinz Wolff Building, Brunel University, Uxbridge, London UB8 3PH, UK.

^3^School of Medicine, Koç Unviersity, Sariyer, Istanbul 34450, Turkey.

^4^Therapeutic Immunology Group, Sir William Dunn School of Pathology, University of Oxford, South Parks Road, Oxford OX1 3RE, UK.

^5^Faculty of Science, Engineering and Computing Kingston University, Penrhyn Road, Kingston upon Thames, Surrey KT1 2EE, UK.

^✍^ Correspondence: Dr Sanjib Bhakta

Email: [s.bhakta@bbk.ac.uk](mailto:s.bhakta@bbk.ac.uk), [sanjib.bhakta@ucl.ac.uk](mailto:sanjib.bhakta@ucl.ac.uk); Tel: +44 (0)20 7631 6355 (Office), +44 (0)20 7079 0799 (Lab); Fax: +44 (0)20 7631 6246.

**Supplementary tables**

**Table S1. List of primers used in this study**

| **Primer** | **Sequence (5’-3’)** | **Experiment used** |
| --- | --- | --- |
| *MSMEG_0305*- *MSMEG_0306*_F | ATGGACCTCAGCGGGTATCT | Operon analysis |
| *MSMEG_0305*- *MSMEG_0306*_R | AACATGTACGCATTCGATCC |  |
| *MSMEG_0306*- *MSMEG_0307*_F | ATGGCGTCGAGCACCTGT |  |
| *MSMEG_0306*- *MSMEG_0307*_R | CGCACGTGCTCACCTACTCT |  |
| *MSMEG_0307*- *MSMEG_0308*_F | TCCCTTCTCATCCATTCCAG |  |
| *MSMEG_0307*- *MSMEG_0308*_R | CCGTCTACTACACCGCTTCG |  |
| *MSMEG_0308*- *MSMEG_0309*_F | GGTGATACCCCTTGATGAGC |  |
| *MSMEG_0308*- *MSMEG_0309*_R | GGTGCATAGCTGACGATGAT |  |
| AraC_pET_F | CGCGCCATATGGTGGAACCGAACG | Cloning of AraC into pET28b(+) |
| AraC_ pET_R | CGCGCGGATCCTCAGTCCCTTCTC |  |
| pMV261araC_F | CGCGC**GGATCC**AGTGGAACCGAACG |  |
| pMVaraC_R | CGCGC**GAATTC**TCAGTCCCTTCTC |  |
| Motif 1_F | GTTGGGGAACGTGTCGAGGCCGTG | Amplification of DNA motifs for EMSA |
| Motif 1_R | CGATCGCGTGCAATGTGCCGAGAT |  |
| Motif 2_F | GCAACCTGGCGGTCCACCGGTCCG |  |
| Motif 2_R | AGTGGAACTCCGAGGCGTAGCTGT |  |
| araC left_F | TTTTTTTTGCATAAATTGCGATGGACCTCAGCGGGTA | Gene deletion |
| araC left_R | TTTTTTTTGCATTTCTTGCTCCACACTCACGTTCACC |  |
| araC right_F | TTTTTTTTTCCATAGATTGGGACTGAATCGTGAAGACCGT |  |
| araC right_R | TTTTTTTTTCCATCTTTTGGGTCGAAGGTCCGCACG |  |
| araC_internal_F | ATCGCGCAGTTCATGCTC | Confirmation of gene deletion |
| araC_internal_R | ACTCCGATATCACCGTGGTC |  |
| araC_ external_F | GTGATCTCACCTGGGTCGGTATCG |  |
| araC_ external_R | GAGCACACCGATGGTCTCGATCA |  |
| msnat-200_F | GCGCAATTGGGACGATGGCAGGCA | Mycobacterial two hybrid system |
| msnat-200_R | GCTGATCGATCCCGGCTCAGGTGTC |  |
| *msaraC*-100_F | CGCGCGGATCCAGTGGAACCGAACG |  |
| *msaraC*-100_R | CGGTCTTATCGATTCAGTCCCTTCTCA |  |
| *MSMEG_0308*-100_F | CGCGCGGATCCAGTGAAGACCGTCT |  |
| *MSMEG_0308*-200_F | CGGGCAATTGTCGTGAAGACCGTCT |  |
| *MSMEG_0308*-100/200_R | CGCGAGATCGATCTAGGTGTGCAGG |  |

**Table S2. Motif 1 presence in the genome of *M. smegmatis*.**

| **Motif Sequence** | ***E*-value** | **Flanking genes** | **EMSA** |
| --- | --- | --- | --- |
| **ACCTC**G**ACAGCAGTTCAGGT** | 0.00045 | *MSMEG_0305/MSMEG_0306* | + |
| **ACCTCAAC**TTTC**GTT**G**AGGT** | 0.0081 | *MSMEG_5819/ MSMEG_5820* | ND |
| **ACCTCAA**GT**G**AG**GTT**G**AGGT** | 0.025 | *MSMEG_0572/MSMEG_0574* | ND |
| **ACCTC**G**AC**C**GC**C**GT**CG**AGG**C | 0.083 | *secF/secD* | ND |
| **ACCTCAAC**G**GC**G**GTT**G**A**CC**T** | 0.14 | *MSMEG_3815/ uvrB* | ND |
| G**CCT**G**AAC**TT**C**CC**TTCAGGT** | 0.29 | *MSMEG_3368/ MSMEG_3369* | ND |
| **ACCTCA**T**CA**TTG**GTT**G**AGGT** | 0.4 | *MSMEG_5661/prrA* | ND |
| **ACCT**GG**ACA**T**C**C**G**AC**C**G**GGT** | 2.2 | *MSMEG_3270/ MSMEG_3271* | ND |
| **ACCT**G**AA**TGA**CA**C**TT**G**AGGT** | 2.7 | *MSMEG_2066/MSMEG_2067* | ND |
| **ACCT**A**AAC**CTT**A**C**TTCAGGT** | 2.7 | *arcA/soxR* | ND |
| **ACCTCAAC**CATT**GTT**G**AGG**G | 3.1 | *MSMEG_3509/ MSMEG_3511* | ND |
| **ACCTC**G**AC**C**G**GTG**T**CGT**GGT** | 3.7 | *MSMEG_6124/ MSMEG_6126* | ND |
| **ACC**G**C**G**AC**C**G**AC**G**CCG**AGGT** | 4.3 | *MSMEG_6452/ MSMEG_6454* | ND |
| **ACC**G**CAAC**G**G**ACC**T**GG**AGGT** | 6.2 | *MSMEG_3140/ MSMEG_3141* | ND |
| **ACCTCAACAG**AT**G**GG**C**T**GGT** | 6.3 | *MSMEG_0542/ MSMEG_0544* | ND |
| **A**A**C**CG**AAC**G**G**TCG**T**CG**AGGT** | 7.2 | *MSMEG_1422/ MSMEG_1423* | ND |
| **ACC**ATG**AC**G**GC**C**GT**CG**AGGT** | 8.8 | *MSMEG_4601/ MSMEG_4602* | ND |
| **ACC**A**C**C**AC**C**G**AC**G**A**TCAGGT** | 8.9 | *MSMEG6702/ MSMEG_6703* | ND |
| **ACC**GAG**AC**G**GCAGTT**GC**GGT** | 9.2 | *MSMEG_0328/ MSMEG_0329* | ND |
| **ACCT**GG**ACAGC**G**G**GG**CA**T**GT** | 9.3 | *MSMEG_6246/ MSMEG_6247* | ND |

**Table S3. Motif 2 presence in the genome of *M. smegmatis*.**

| **Motif Sequence** | ***E*-value** | **Flanking genes** | **EMSA** | |
| --- | --- | --- | --- | --- |
| **AG**C**A**A**GAAA**A**G**T**CAT**G**TCCT**GAC | 0.0028 | *MSMEG_0307/ MSMEG_0308* | | - |
| TCCGC**GAA**T**T**C**ACAT**G**T**TT**TTCT** | 1.6 | *MSMEG_5557/MSMEG_5558* | | ND |
| T**GAAG**A**AAT**CGTCGAGTTCCTGA | 3.4 | *MSMEG_6243/ MSMEG_6244* | | ND |
| **A**CC**AGGA**GT**T**CT**CAT**G**TCC**CAGA | 5.4 | *MSMEG_0621/ espG3* | | ND |
| T**GA**G**G**TG**AATGACA**A**GT**A**CTT**GA | 9 | *MSMEG_0295/ MSMEG_0296* | | ND |

**Table S4. Bacterial strains and plasmids used in this study.**

| Strain or plasmid | Characteristics | Reference |
| --- | --- | --- |
| **Strains** |  |  |
| *M. smegmatis* mc^2^155 | Efficient plasmid transformation (Ept) | [[19](#_ENREF_19)] |
| Δ *nat* | *M. smegmatis* mc^2^155 with the nat *MSMEG_0306* gene replaced by a Kan^R^ marker | [[35](#_ENREF_35)] |
|  |  |  |
| Δ *MSMEG_0307* | *M. smegmatis* mc^2^155 with the *MSMEG_0307* gene replaced by a Hyg^R^ marker | This study |
| Δ *MSMEG_0308* | *M. smegmatis* mc^2^155 with the *MSMEG_0308* gene replaced by a Hyg^R^ marker | This study |
| *M. smegmatis* mc^2^155-pMV261 | *M. smegmatis* mc^2^155 with the empty pMV261 plasmid | This study |
| *M. smegmatis* mc^2^155-pMV*araC* | *M. smegmatis* mc^2^155 overexpressing the *MSMEG_0307* gene | This study |
| Δ *MSMEG_0307*-pMV*araC* | *M. smegmatis* mc^2^155 with the *MSMEG_0307* gene replaced by a Hyg^R^ marker and overexpressed via the pMV*araC* plasmid | This study |
| *E. coli* BL21(DE3)pLysS | Overexpression strain; F-, *omp*T, *hsd*SB, (r_B_^-^, m_B_^-^), *dcm*, *gal*, λ(DE3), pLysS, *cm*^R^ | New England Biolabs Inc. (www.neb.com) |
| *E. coli* BL21(DE3)pLysS::pET28b(+)*MSMEG_0307* | Genetically modified *E. coli* strain for the heterologous expression  of the MSMEG_0307 protein | This study |
| **Phages** |  |  |
| phAE159 | Conditionally replicating shuttle phasmid derived from the lytic mycobacteriophage TM4 | [[27](#_ENREF_27)] |
| ph*Δ* *MSMEG_0307* | Derivative of the phAE159 containing the p0004S*araC* on its unique *Pac*I site | This study |
| **Plasmids** |  |  |
| pET28b(+) | Expression vector producing hexa-histidine-tagged recombinant proteins | Novagen  (www.novagen.com) |
| pET28b(+)*MSMEG_0307* | Expression vector producing His-tagged MSMEG_0307 protein | This study |
| p0004s | Vector for cloning allelic-exchange substrates to be used for specialized transduction; contains λ phage *cos* site and Hyg^R^ marker | Gift from W. R. Jacobs Jr |
| p0004S *MSMEG_0307* | Derivative of the p0004S containing the genetic material for the allelic exchange of the *MSMEG_0307* gene | This study |
| pMV261 | Shuttle-vector capable of replication in both E. coli and mycobacteria.  The expression of the cloned gene is under the control of the *hsp60* mycobacterial promoter. | [[28](#_ENREF_28)] |
| pMV*araC* | pMV261 with the MSMEG_0308 gene cloned | This study |
| pAUB100 | Episomal shuttle plasmid used for mycobacterial protein-protein interaction studies; “preys” constructs were cloned into this plasmid | [[42](#_ENREF_42)] |
| pAUB200 | Integrative shuttle plasmid used for mycobacterial protein-protein interaction studies; “baits” constructs were cloned into this plasmid | [[42](#_ENREF_42)] |
| pAUB100 *MSMEG_0307* | Derivative of pAUB100 containing the *MSMEG_0307* gen | This study |
| pAUB100 *MSMEG_0308* | Derivative of pAUB100 containing the *MSMEG_0308* gene (*MSMEG_0308*) | This study |
| pAUB200*nat* | Derivative of pAUB100 containing the *nat* gene (*MSMEG_0306*) | This study |
| pAUB200 *MSMEG_0308* | Derivative of pAUB100 containing the *MSMEG_0308* gene (*MSMEG_0308*) | This study |

**Table S4. Comparison of the differential gene expression responses of the hypothetical MSMEG_0607 regulon genes in M. smegmatis mc2155 using publicly available microarray data.** Overall the genes that contain the motif 1 in their intergenic region possess similar transcriptomic signatures apart from specific discrepancies found in some of the genes when the values of three publicly available microarray data were compared. Supplementary Excel file mmc1.

**Supplementary figures**

slow- growing

*M. tuberculosis*

*M. bovis BCG*

*M. avium*

*M. ulcerans/*

*M. marinum*

fast- growing

*M. smegmatis*

*M. vanbaalenii*

*M. gilvum*

*M. abcessus*

*hsaA*

*hsaD*

*hsaC*

*hsaB*

*pseudogene*

*nat*

*araC*

*reductase*

*aspB*

*hypothetical genes*

*dehydrogenase homologues*

***hsaA***

***hsaD***

***hsaC***

***hsaB***

***nat***

***nat***

***araC***

***reductase***

*Rv3566c*

*Rv3567c*

*Rv3568c*

*Rv3569c*

*Rv3570c*

*Mb3596c*

*Mb3598c*

*Mb3599c*

*Mb3600c*

*Mb3601c*

*MAV_0595*

*MAV_0594*

*MAV_0591*

*MAV_0592*

*MAV_0593*

*MMAR_5054*

*MMAR_5055*

*MMAR_5056*

*MSMEG_0306*

*MSMEG_0307*

*MSMEG_0307*

*Mvan_2350*

*Mvan_2351*

*Mvan_2352*

*Mvan_2353*

*Mvan_2354*

*Mflv_0433*

*Mflv_0432*

*Mflv_0431*

*Mflv_0430*

*Mflv_0429*

*MASS_3981*

*MASS_3982*

*MASS_3983*

*MASS_3984*

**Figure S1. Comparison of the *nat* operon in mycobacteria**. The genes are annotated by colour. Genome sequences were obtained from NCBI web server and the operon organisation was analysed using Artemis and ACT softwares.


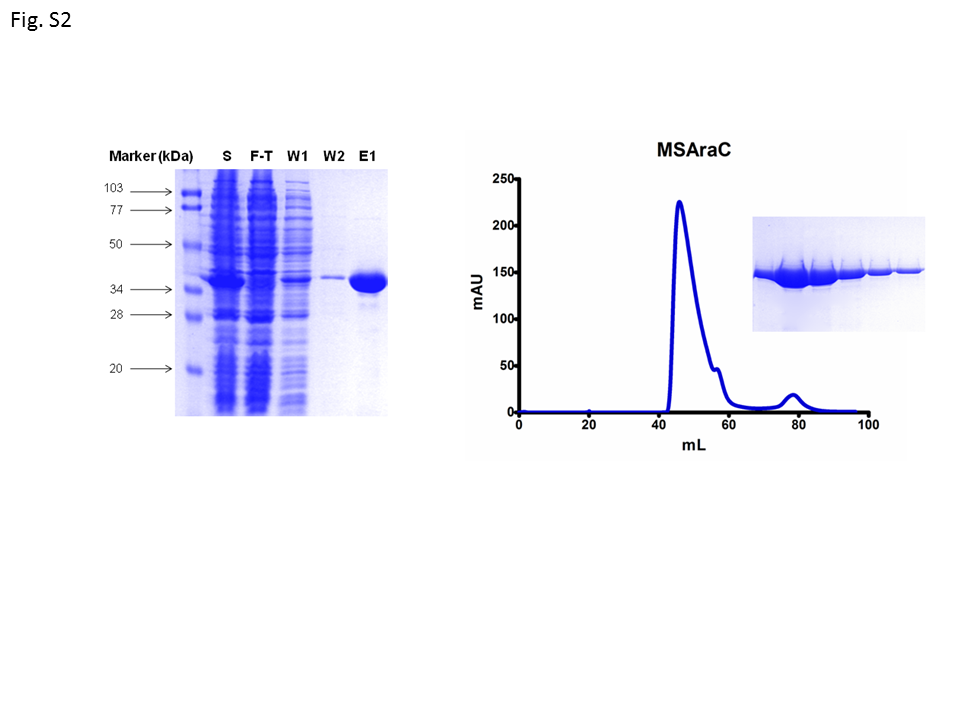


**Figure S2. Purification of recombinant MSMEG_0307.** *E. coli* BL21(DE3)pLysS cells containing the pET28b(+)*MSMEG_0307* plasmid were grown, induced and lysed as described in Materials and methods. (A) SDS-PAGE analysis of the purification fractions (10 μL per lane). The gel was stained with Coomassie blue. Protein markers are low-range molecular marker (Biorad); S, is the soluble protein lysate. F-T is the flow-through of the lysate after passing by a 5 mL Ni-NTA column. W1 is the fraction following a 20 column volume wash with Tris buffer containing 60 mM Imidazole (IMZ). W2 is the fraction following a 20 column volume wash with Tris buffer containing 120 mM IMZ. Finally E1 is the eluted protein in buffer containing 250 mM IMZ. (B) Purified by Ni-NTA **MSMEG_0307** proteins were subjected to a HiLoad 16/60 Superdex™ 75 pg (Pharmacia) gel filtration column, equilibrated with 20 mM Tris.HCl, pH 8, 100 mM NaCl buffer, using an Äkta purifier (GE Healthcare). mAU represents the A280nm. Protein peak correspond to pure (>99%) proteins. SDS-PAGE analysis of the peak fractions are depicted in the figures in frame.

_
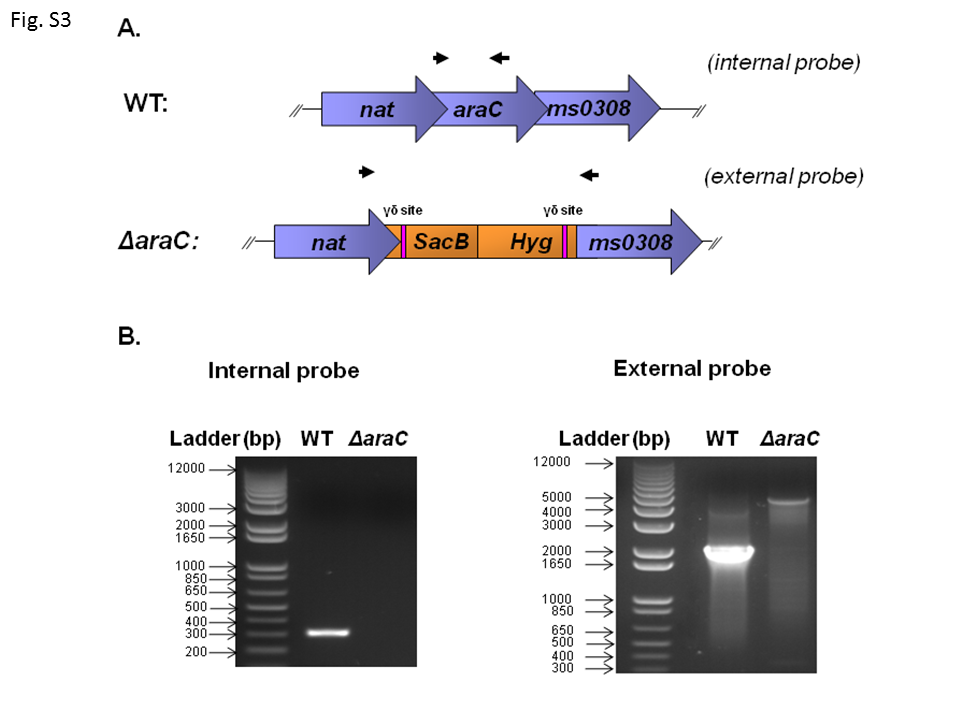
_

**Figure S3. PCR confirmation of *ΔaraC* mutant**. (A) Schematic representation of the confirmation of the *ΔMSMEG_0307* strain by PCR. Broad arrows represent the gene organisation and arrowheads show the sites where the forward and reverse primers anneal. Internal probes bind within the *araC* gene whereas the external set binds in the flanking regions of the *MSMEG_0307*gene, i.e. the *nat* and the *MSMEG_0308* gene. (B) PCR amplification using the internal and external primers on gDNA purified from WT and *ΔMSMEG_0307* *M. smegmatis*. The external PCR product of the *ΔMSMEG_0307* was gel extracted and sequenced for further confirmation.

**
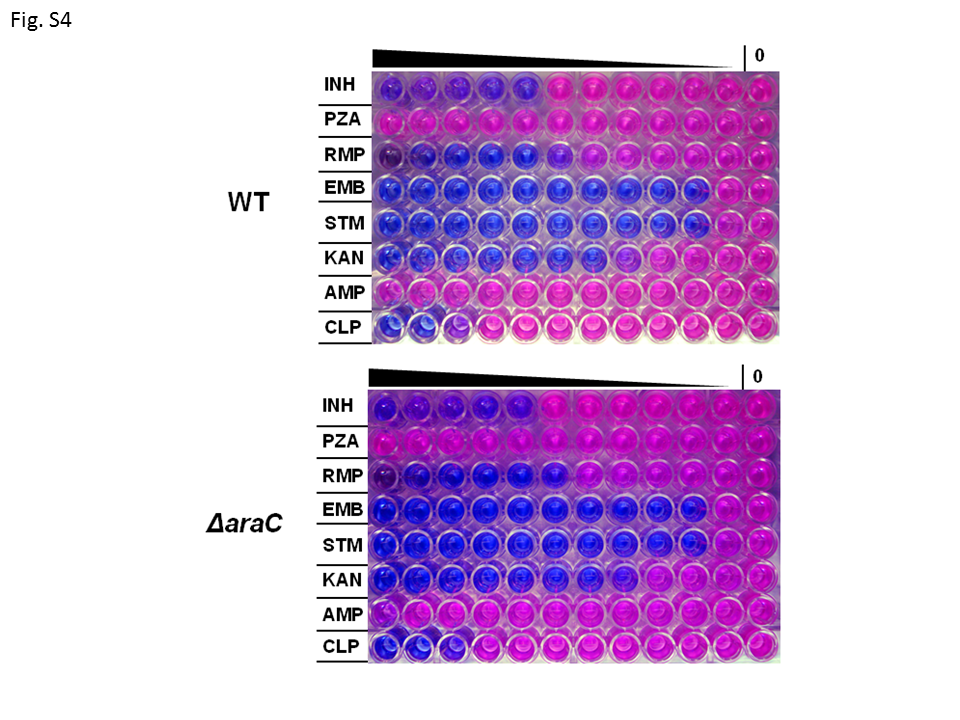
**

**Figure S4. Antibiotic susceptibility of WT and *Δ****MSMEG_0307* ***M. smegmatis***. Antibiotics were serially diluted by 2 fold across the length of the plate. The right hand column was with no drug (positive control). Antibiotics that were used and their concentrations in μg/ mL are: isoniazid (INH 50 to 0.09), pyrazinamide (PZA 50 to 0.09), rifampicin (RMP 50 to 0.09), ethambutol (EMB 150 to 0.29), streptomycin (STM 50 to 0.09), kanamycin (KAN 50 to 0.09), Ampicilin (AMP 150 to 0.29) and chloramphenicol (CLP 50 to 0.09). The wedges denote the decrease in antibiotic concentration.

**Figure S5. Protein-protein interactions**. Protein-protein interactions were investigated using the mycobacterial protein fragment complementation assay as previously described [[42](#_ENREF_42)] with some modifications. Briefly, the genes encoding the NAT (*MSMEG_0306*) and MSMEG_0308 (*MSMEG_0308*) proteins were cloned into the pAUB200 vector and used as ‘‘bait’’. Similarly, the genes encoding the AraC-family transcriptional regulator MSMEG_0307AraC and MSMEG_0308 proteins (*MSMEG_0307*, *MSMEG_0308*) were cloned into the pAUB100 vector and were used as the ‘‘prey’’. A combination of ‘‘bait’’ and ‘‘prey’’ sets were introduced into *M. smegmatis* mc^2^155 using electroporation. Protein-protein interactions were identified by the growth of *M. smegmatis* mc^2^155 cells possessing a set of ‘‘bait’’ and ‘‘prey’’ on Middlebrook 7H11 agar containing 12 µg mL^-1^ trimethoprim. Cells were streaked on 7H10 plates and incubated for 5 days at 37°C. Pictures represent a part of the plate and were taken using the Gel-Doc system.
